# Supplementary material for: Long COVID and financial outcomes: evidence from four longitudinal population surveys
Source: J Epidemiol Community Health. 2024 Mar 20;78(7):458–65. doi: 10.1136/jech-2023-221059 (PMC11187380; doi:10.1136/jech-2023-221059)
Supplement: Supplementary data [file jech-2023-221059supp001.pdf]

Supplementary Material

Supplementary 1) Potential confounders and Directed Acyclic Graph 2
Supplementary 2) Exposure and outcomes by cohort...5
Supplementary 3) New benefit claims but COVID status 6
Supplementary 4) Sensitivity analyses using imputed data 7
Supplementary 5) Sensitivity analysis using a four-category measure of long-COVID & stratified analysis 8
Supplementary 6) OECD equivalised measure of household income 9
Supplementary 7) Re-weighted analysis using scaled populations weights 10

Rhead R, *et al. J Epidemiol Community Health* 2024; 78:458–465. doi: 10.1136/jech-2023-221059

Supplementary 1) Potential confounders and Directed Acyclic Graph

Characteristic	Overall = 20112[1]	NCDS N = 6467[1]	BCS70 N = 5421[1]	NS N = 4005[1]	MCS N = 4219[1]
**Sex**					
Male	8,460 (42.2%)	2,995 (46.3%)	2,293 (42.3%)	1,501 (38.0%)	1,671 (40.1%)
Female	11,652 (57.8%)	3,472 (53.7%)	3,128 (57.7%)	2,504 (62.0%)	2,548 (59.9%)
**Ethnicity**					
White	18,162 (94.7%)	6,467 (100.0%)	5,421 (100.0%)	2,941 (90.3%)	3,333 (85.2%)
Non-White	1,950 (5.3%)	0 (0.0%)	0 (0.0%)	1,064 (9.7%)	886 (14.8%)
**Pre-pandemic employment**					
Employed	10,781 (61.8%)	3,238 (54.9%)	3,967 (86.7%)	2,718 (84.9%)	858 (25.0%)
Unemployed	541 (2.9%)	125 (2.1%)	93 (2.0%)	133 (3.2%)	190 (4.6%)
Economically Inactive	6,157 (35.3%)	2,535 (43.0%)	518 (11.3%)	388 (11.8%)	2,716 (70.4%)
Unknown	2,680	569	843	792	476
**Shielding during the pandemic**					
No	19,023 (94.7%)	5,958 (92.2%)	5,108 (94.2%)	3,855 (96.4%)	4,102 (97.2%)
Yes	1,081 (5.3%)	506 (7.8%)	312 (5.8%)	150 (3.6%)	113 (2.8%)
Unknown	8	3	1	0	4
**Keyworker during the pandemic**					
No	12,521 (65.6%)	4,422 (72.1%)	2,709 (52.9%)	2,019 (52.5%)	3,371 (83.2%)
Yes	6,515 (34.4%)	1,708 (27.9%)	2,409 (47.1%)	1,748 (47.5%)	650 (16.8%)
Unknown	1,127	337	303	254	233
**Pre-pandemic education (NVQ)**					
None	990 (5.5%)	383 (6.0%)	363 (7.1%)	113 (3.2%)	131 (4.6%)
NVQ1 level	1,247 (7.3%)	602 (9.4%)	349 (6.8%)	199 (5.6%)	97 (4.9%)
NVQ2 level	4,067 (24.2%)	1,567 (24.5%)	1,322 (25.9%)	714 (20.6%)	464 (25.3%)
NVQ3 level	3,026 (17.8%)	1,154 (18.1%)	753 (14.8%)	824 (22.7%)	295 (15.5%)
NVQ4 level	6,084 (36.3%)	2,349 (36.7%)	1,878 (36.9%)	1,100 (30.9%)	757 (43.5%)
NVQ5 level	1,540 (9.0%)	337 (5.3%)	431 (8.5%)	664 (17.1%)	108 (6.2%)
Unknown	3,351	75	325	405	2,545
**Chronic health condition (pre-pandemic)**					
No	13,586 (72.9%)	4,122 (68.1%)	3,161 (64.6%)	2,932 (80.0%)	3,371 (82.2%)
Yes	5,072 (27.1%)	1,934 (31.9%)	1,730 (35.4%)	686 (20.0%)	722 (17.8%)
Unknown	1,477	411	530	402	134
**Probable psychological distress (pre-pandemic)**					
No	15,268 (82.7%)	5,218 (87.2%)	4,086 (83.5%)	2,627 (74.6%)	3,337 (82.8%)
Yes	3,212 (17.3%)	769 (12.8%)	805 (16.5%)	919 (25.4%)	719 (17.2%)
Unknown	1,654	480	530	464	180

[1]n_unweighted (%)

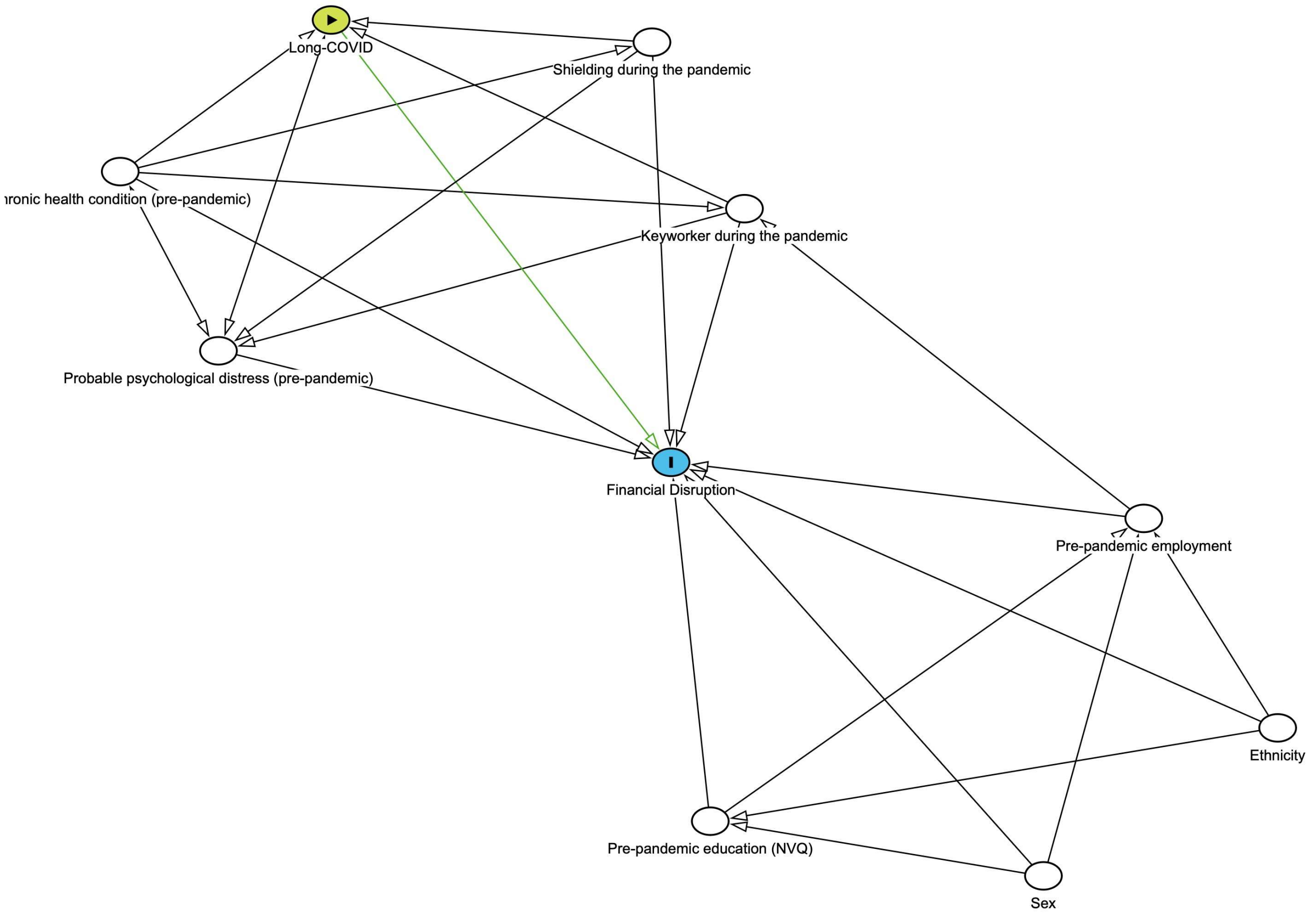

Directed acyclic graph (DAG) to illustrate confounders relationship with exposure and outcomes

Supplementary 2) Exposure and outcomes by cohort

Characteristic	Overall<br>N = 20,112[1]	NCDS<br>N = 6,467[1]	BCS70<br>N = 5,421[1]	NS<br>N = 4,005[1]	MCS<br>N = 4,219[1]
**Long COVID**					
No COVID	16,731 (83.1%)	5,783 (89.4%)	4,565 (84.2%)	3,190 (80.6%)	3,193 (75.3%)
C19 - normal functioning	879 (4.5%)	133 (2.1%)	192 (3.5%)	194 (4.7%)	360 (8.7%)
C19 - sym <4weeks	2,139 (10.7%)	426 (6.6%)	537 (9.9%)	550 (12.9%)	626 (15.1%)
C19 - sym 4-<12 weeks	251 (1.2%)	92 (1.4%)	82 (1.5%)	49 (1.1%)	28 (0.7%)
C19 - sym 12+ weeks	112 (0.6%)	33 (0.5%)	45 (0.8%)	22 (0.6%)	12 (0.3%)
**Coping financially compared to pre-pandemic**					
Same/better	14,585 (72.8%)	4,740 (73.3%)	3,940 (72.7%)	2,932 (73.7%)	2,973 (71.2%)
Worse	5,527 (27.2%)	1,727 (26.7%)	1,481 (27.3%)	1,073 (26.3%)	1,246 (28.8%)
**New benefit claims since pandemic**					
No	17,303 (86.3%)	5,637 (87.2%)	4,631 (85.4%)	3,356 (84.1%)	3,679 (88.0%)
Yes	2,809 (13.7%)	830 (12.8%)	790 (14.6%)	649 (15.9%)	540 (12.0%)
**Change in weekly household income since pandemic**					
same/increased & decrease < 5%	12,662 (81.9%)	3,918 (82.1%)	3,453 (81.4%)	2,843 (82.8%)	2,448 (81.2%)
decreased by ≥ 5%	2,733 (18.1%)	856 (17.9%)	787 (18.6%)	561 (17.2%)	529 (18.8%)
Unknown	4,760	1,693	1,181	583	1,303

[1] unweighted n (%). NCDS refers to the 1958 National Child Development Study; BCS70 refers to the 1970 British Cohort study; NS refers to the 1989-90 Next Step study; MCS refers to the 2000-01 Millennium Cohort Study.

Rhead R, *et al. J Epidemiol Community Health* 2024; 78:458–465. doi: 10.1136/jech-2023-221059

Supplementary 3) New benefit claims but COVID status

	Overall = 20112[1]	no covid N = 16731[1]	C19 - normal functioning N = 879[1]	C19 - sym <4weeks N = 2139[1]	C19 - 4+ weeks N = 363[1]
**New benefit claims?**					
No	17,256	14,410 (83.33%)	770 (4.59%)	1,780 (10.37%)	296 (1.70%)
Yes	2,856	2,321 (81.52%)	109 (3.71%)	359 (12.43%)	67 (2.34%)
[1]n_unweighted (%)					

	Free school dinners	Universal credit	Employment support	Sick pay	Council tax support	COVID-19 self-employment income support	Career allowance	Test and trace
No COVID	79 (3.4%)	873 (37.6%)	249 (10.7%)	199 (8.6%)	281 (12.1%)	771 (33.2%)	302 (13.0%)	32 (1.4%)
C19 - normal functioning	4 (3.7%)	41 (37.6%)	7 (6.4%)	17 (15.6%)	11 (10.1%)	31 (28.4%)	4 (3.7%)	9 (8.3%)
C19 - symptoms	14 (3.3%)	157 (36.9%)	39 (9.2%)	72 (16.9%)	56 (13.1%)	108 (25.4%)	40 (9.4%)	32 (7.5%)

	Free school dinners	Universal credit	Employment support	Sick pay	Council tax support	COVID-19 self-employment income support	Career allowance	Test and trace
No COVID	79 (3.4%)	873 (37.6%)	249 (10.7%)	199 (8.6%)	281 (12.1%)	771 (33.2%)	302 (13.0%)	32 (1.4%)
C19 - normal functioning	4 (3.7%)	41 (37.6%)	7 (6.4%)	17 (15.6%)	11 (10.1%)	31 (28.4%)	4 (3.7%)	9 (8.3%)
C19 - sym <4weeks	13 (3.6%)	135 (37.6%)	32 (8.9%)	54 (15.0%)	46 (12.8%)	99 (27.6%)	27 (7.5%)	26 (7.2%)
C19 - sym 4-<12 weeks	1 (2.6%)	12 (30.8%)	4 (10.3%)	10 (25.6%)	5 (12.8%)	4 (10.3%)	8 (20.5%)	5 (12.8%)
C19 - sym 12+ weeks		10 (35.7%)	3 (10.7%)	8 (28.6%)	5 (17.9%)	5 (17.9%)	5 (17.9%)	1 (3.6%)

Supplementary 4) Sensitivity analyses using imputed data

Long-COVID associated with subject of financial well-being and new benefit claims (complete case and MI)

Long-COVID	Crude		Adjusted		Adjusted (MICE imputed data)
	n	RRR (95% CI)	n	RRR (95% CI)	RRR (95% CI)
**Financial wellbeing**					
no covid	16,731	--	11,323	--	--
C19 - normal functioning	879	0.93 (0.82, 1.06)	505	1.02 (0.86, 1.21)	0.96 (0.85, 1.09)
C19 - symptoms <4weeks	2,139	1.11 (1.03, 1.20)	1,326	1.15 (1.04, 1.26)	1.13 (1.05, 1.22)
C19 - symptoms 4-<12 weeks	251	1.29 (1.08, 1.55)	176	1.28 (1.01, 1.62)	1.28 (1.07, 1.54)
C19 - symptoms 12+ weeks	112	1.57 (1.25, 1.96)	78	1.85 (1.43, 2.41)	1.62 (1.30, 2.03)
**New benefit claims**					
no covid	16,731	--	14,314	--	--
C19 - normal functioning	879	0.85 (0.69, 1.04)	638	0.94 (0.72, 1.22)	0.92 (0.75, 1.13)
C19 - symptoms <4weeks	2,139	1.19 (1.06, 1.33)	1,670	1.34 (1.17, 1.53)	1.21 (1.08, 1.35)
C19 - symptoms 4-<12 weeks	251	1.12 (0.83, 1.51)	233	1.16 (0.83, 1.63)	1.04 (0.78, 1.40)
C19 - symptoms 12+ weeks	112	1.79 (1.27, 2.53)	99	1.79 (1.20, 2.66)	1.71 (1.22, 2.39)

Long-COVID associated with a decrease in weekly household income ≥ 5% (complete case and MI)

Long-COVID	Crude	Adjusted
	RRR (95% CI)	RRR (95% CI)
**Decrease in weekly household income ≥ 5% (complete case)**		
C19 - normal functioning	0.91 (0.79, 1.05)	1.03 (0.85, 1.23)
C19 - symptoms <4weeks	1.00 (0.91, 1.09)	1.05 (0.93, 1.18)
C19 - symptoms 4+ weeks	1.07 (0.88, 1.30)	1.25 (1.02, 1.53)
**Decrease in weekly household income ≥ 5% (missing data imputed)**		
C19 - normal functioning	1.01 (0.95, 1.08)	1.02 (0.95, 1.09)
C19 - symptoms <4weeks	1.01 (0.96, 1.07)	1.02 (0.97, 1.07)
C19 - symptoms 4+ weeks	1.05 (0.97, 1.14)	1.07 (0.99, 1.15)

Rhead R, *et al. J Epidemiol Community Health* 2024; 78:458–465. doi: 10.1136/jech-2023-221059

Supplementary 5) Sensitivity analysis using a four-category measure of long-COVID & stratified analysis

Long-COVID (four-category) associated with financial wellbeing

Long-COVID	Financial wellbeing	
	Crude	Adjusted
	RRR (95% CI)	RRR (95% CI)
C19 - normal functioning	0.93 (0.82, 1.06)	0.95 (0.81, 1.11)
C19 - symptoms <4weeks	1.11 (1.03, 1.20)	1.11 (1.02, 1.21)
C19 - symptoms 4+weeks	1.38 (1.19, 1.59)	1.32 (1.12, 1.56)

Long-COVID (four-category) associated with new benefit claims

Long-COVID	New benefit claims	
	Crude	Adjusted
	RRR (95% CI)	RRR (95% CI)
C19 - normal functioning	0.85 (0.69, 1.04)	0.94 (0.72, 1.22)
C19 - symptoms <4weeks	1.19 (1.06, 1.33)	1.34 (1.17, 1.53)
C19 - symptoms 4+weeks	1.33 (1.06, 1.68)	1.36 (1.05, 1.77)

Long-COVID associated with subjective financial well-being, stratified by sex

Financial wellbeing						
Long-COVID	Male			Female		
	n	RRR	95% CI	n	RRR	95% CI
no covid	7,362	—	—	9,971	—	—
C19 - normal functioning	462	0.83	0.68, 1.02	469	1.04	0.88, 1.21
C19 - symptoms <4weeks	851	1.10	0.97, 1.24	1,372	1.12	1.02, 1.23
C19 - symptoms 4-<12 weeks	77	1.91	1.50, 2.42	178	1.02	0.80, 1.32
C19 - symptoms 12+ weeks	43	1.58	1.07, 2.32	74	1.56	1.18, 2.05

Supplementary 6) OECD equivalised measure of household income

Both pandemic and pre-pandemic income measures were equivalised using the Organisation for Economic Co-operation and Development (OECD) equivalence scale where household income was divided by the square root of the household size. This implies that, for example, the needs of a household of four are twice as large as one composed of a single person. Equivalised income measures were then log-transformed to account for the skewed distribution of data. Linear regression was used to examine the association between long COVID and change in weekly household income.

Additional analysis of the associations between OECD equivalized weekly household income (adjusting for retrospective pre-pandemic income) and a four-category measure of COVID severity are shown in Figure a. Findings support those reported in Figure 3, suggesting that those with COVID-19 symptoms which last longer than 4 weeks have decreased household income (adjusted β=-0.05, CI=-0.10, -0.01).

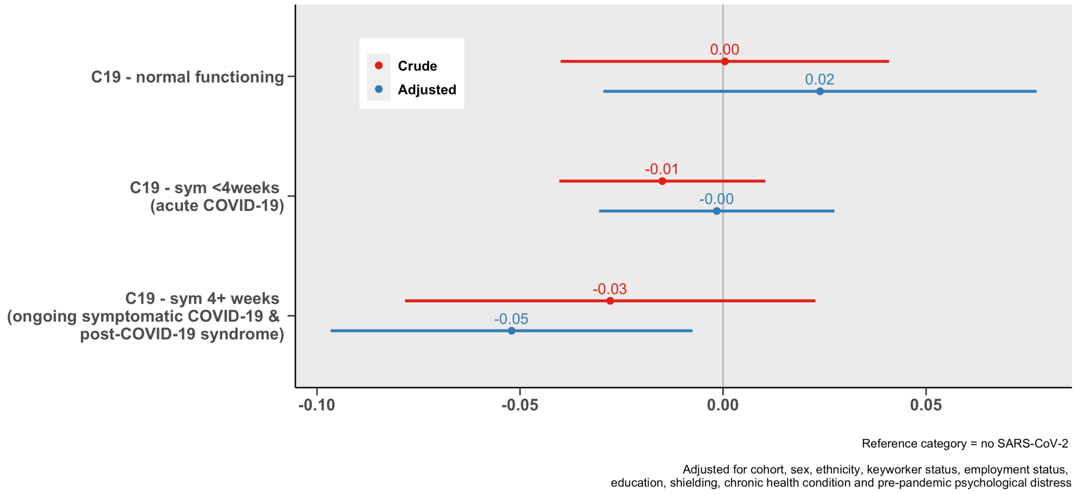

Figure a: Association between duration of COVID-19 symptoms and change in weekly household income across four CLS cohorts

Supplementary 7) Re-weighted analysis using scaled populations weights

Long-COVID associated with subject of financial well-being, new benefit claims and change in weekly household income (population age composite weights)

	Crude estimates using population age composition weights
**Financial coping**	**RRR (95% CI)**
C19 - normal functioning	0.93 (0.82, 1.06)
C19 - symptoms <4weeks	1.11 (1.03, 1.20)
C19 - symptoms 4-<12 weeks	1.29 (1.06, 1.56)
C19 - symptoms 12+ weeks	1.52 (1.20, 1.93)
**New benefit claims**	
C19 - normal functioning	0.83 (0.67, 1.03)
C19 - symptoms <4weeks	1.19 (1.06, 1.34)
C19 - symptoms 4-<12 weeks	1.16 (0.85, 1.59)
C19 - symptoms 12+ weeks	1.84 (1.29, 2.62)
**Decrease in weekly household income ≥ 5%**	
C19 - normal functioning	0.92 (0.79, 1.06)
C19 - symptoms <4weeks	1.00 (0.91, 1.10)
C19 - symptoms 4+ weeks	1.04 (0.84, 1.29)

Rhead R, *et al. J Epidemiol Community Health* 2024; 78:458–465. doi: 10.1136/jech-2023-221059
